# Surgical and anesthetic considerations of laryngeal saccular cyst: a case report

**DOI:** 10.1186/1752-1947-5-283

**Published:** 2011-07-05

**Authors:** Ioannis Aidonis, Anargyros Skalimis, Efthimios Kirodimos

**Affiliations:** 1 ENT Department, Euromedica Kyanous Stavros General Hospital, Vizyis & Vyzantos 1, Thessaloniki 54636, Greece; 2 Anesthetic Department, Euromedica Kyanous Stavros General Hospital, Vizyis & Vyzantos 1, Thessaloniki 54636, Greece; 3 ENT Department, Hippokration Hospital, Athens Medical School, Athens, Greece

## Abstract

**Introduction:**

Supraglottic laryngeal cysts are benign, uncommon lesions that have the potential to cause airway compromise.

**Case Presentation:**

We present a case of a 46-year-old Caucasian woman who was scheduled for excision of a large neck growth (saccular cyst) and was managed successfully. There was thorough consideration regarding anesthetic and surgical management. Steps taken led to a successful excision with no recurrence during follow up.

**Conclusion:**

This case was an opportunity to consider the challenges in the airway management associated with such cysts and provided reassurance that excision of these cysts is associated with a good post-operative outcome.

## Introduction

The presence of a large neck growth can potentially cause difficulty during intubation in theatre as well as in the emergency department. Supraglottic laryngeal cysts such as saccular ones may be benign, and uncommon, lesions. On rare occasions, they have been reported to cause life-threatening events through acute airway obstruction. However, when proper assessment has taken place in the pre-operative stage and the anesthetic and surgical management have been well planned, complications and morbidity can be avoided. In our case, our patient was assessed pre-operatively, and both her anesthetic and surgical management ensured her well-being.

## Case presentation

A 46-year-old Caucasian woman was referred to our clinic with a 12-month history of hoarseness and progressive difficulty in breathing. Weeks prior to surgery she developed minor dysphagia which, in combination with the earlier symptoms, convinced her to seek medical advice and treatment. Past medical history included smoking (20 cigarettes per day), with no other significant co-morbidity.

**Figure 1:**
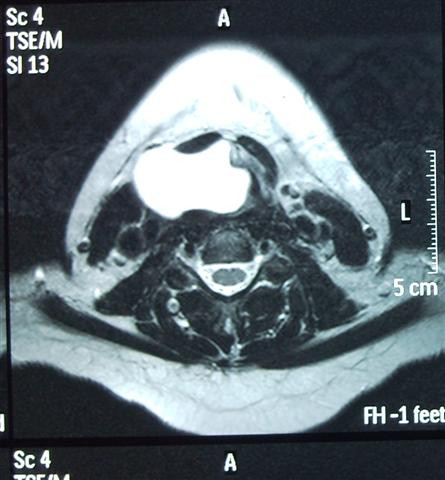
**Sagittal T2-weighted MRI image of the neck demonstrating a well defined lesion**.

**Figure 2:**
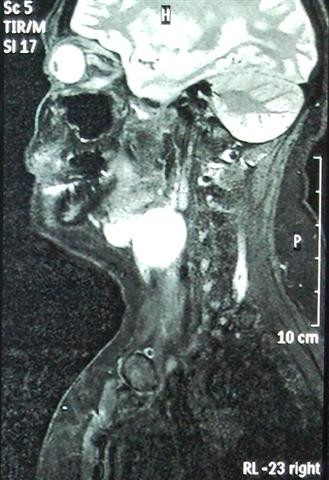
**Axial T2-weighted MRI image showing a large lesion partially obstructing the supraglottic airway structures**.

On examination, she had an easily recognized right neck mass, which in palpation felt soft, mobile and non-pulsatile. There were no lymph nodes or other masses nearby. She also had a noticeable breathy dysphonia. On flexible nasendoscopy, there was a large well-defined supraglottic lesion partially obstructing her vocal cords, which were anatomically and functionally intact. An urgent neck computed tomography (CT) scan was ordered, which revealed an extensive cystic supraglottic mass with no further anomalies of the larynx (Figures 1 and 2). She was urgently scheduled for surgical management. Her pre-operative tests were of no significance. On airway assessment, Mallampati score was found to be (II), her jaw movement was not limited and thyromental distance was more than three finger breadths. Nevertheless, a fiberoptic intubation kit was arranged as back-up for the induction and the whole procedure.

On the day of the operation, our patient had no premedication. The plan was to induce our patient and test the ability to bag-mask ventilate before administration of a muscle relaxant. The option of awake intubation and gaseous induction was offered, however our patient refused it. An emergency tracheotomy kit was readily available as a last option. Following pre-oxygenation, midazolam 2 mg, fentanyl 100 μg and propofol 200 mg were administered and manual ventilation ability was assessed. It proved to be feasible and subsequently suxamethonium 100 mg was given. Conventional laryngoscopy was attempted at that stage that showed a Cormack and Lehane view Grade IV. Due to the readily available equipment we proceeded down the fiberoptic intubation route. The oral route was chosen to avoid extra tissue trauma that could affect visibility. The initial view was that of a mass obstructing all the structures in the supraglottic area. There was a need for manipulation with the tip of the scope to bypass it as non-traumatically as possible in order to get a view of the vocal cords. Insertion of the tube (flexible size 7.0id) was uneventful. As a result, her airway was secured and we proceeded to the operation. Maintenance of anesthesia was achieved with an oxygen and nitrous oxide mix (65% to 35% respectively) and sevoflurane 1.9%.

**Figure 3:**
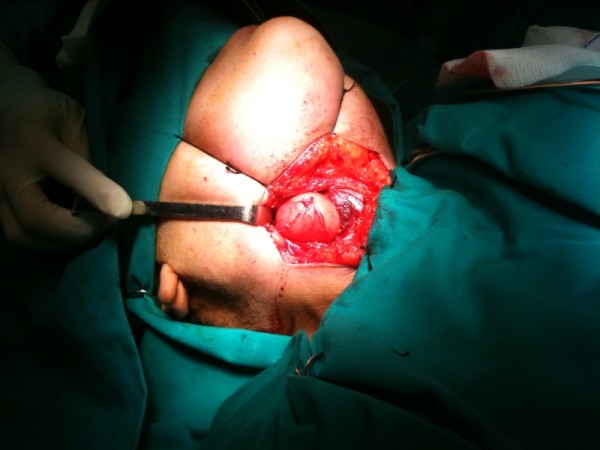
**Intra-operative picture revealing the cyst viewed via the external approach**.

The mass was excised with an external approach (Figure 3). This method was preferable to a laser endoscopic vestibulectomy, as it appears to be more efficient in the management of huge cysts [1]. No intra-operative difficulties were faced. At the end of the procedure and just before extubation, conventional laryngoscopy was performed both to confirm airway patency and facilitate suctioning under direct view. The Cormack and Lehane grade this time was Grade I, which was very reassuring for the extubation and recovery period. Both were uneventful.

Inspection of the lesion revealed a brownish soft mucosal lined sac sized 5.5 × 2.9 × 4 cm which contained mucus. A histopathology report confirmed the diagnosis of a cyst lined with pseudostratified columnar respiratory epithelium and focal presence of intracellular and extracellular mucus production (saccular laryngeal cyst). No evidence of cancer cells in the specimen was found. At follow-up eight months later there was no recurrence.

## Discussion

Supraglottic laryngeal cysts are benign and relatively rare formations that originate from the laryngeal saccule and can mimic other types of more common laryngeal anomalies [2]. This saccule is an opening located in the roof of the laryngeal ventricle. Its role is in lubrication of the vibrating vocal fold with mucus produced from the specifically designed epithelium. Continuous secretion of this mucus in a blocked laryngeal saccule opening is the main cause of saccular cyst formation [2]. Its size can increase as it expands gradually and spreads through the thyrohyoid membrane into the neck, applying pressure to neighboring anatomical structures. Left undiagnosed and neglected these effects may be detrimental, leading to life-threatening acute airway obstruction [3,4]. Therefore, appropriate and meticulous anesthetic management is of utmost importance.

Detailed history prior to any anesthetic intervention is vital. Signs and symptoms such as hoarseness, neck swelling, stridor, dysphagia, globus, sore throat, snoring and cough can alert the anesthetist to potential difficulties with intubation and ventilation, especially after the administration of muscle relaxants. Imaging studies may contribute by revealing the size of the lesion and its anatomical relations. If there is cause for concern, preparation should be made for management of a difficult airway. A person skilled in fiberoptic intubation should be immediately available. Maintenance of oxygenation via cricothyrotomy, tracheotomy or even cardio-pulmonary bypass may be required in extreme situations [5]. In the majority of cases, at the end of the operation structures are better defined and extubation can be less eventful than intubation [1].

As far as surgical management is concerned, these entities can be efficiently managed with endoscopy when they are diagnosed at the early stages of the disease [6]. However, the external approach can be safer in neglected cases that present as large masses. This approach provides better visualization and exposure conditions [7]. With this technique, the cyst is excised via a lateral cervical approach through the thyrohyoid membrane. This can avoid the damage to the neurovascular bundle that is more common in endoscopic surgery [8]. Another advantage of external approach is the lower recurrence rate compared to endoscopic surgery [7,8].

A useful tool for selecting the ideal approach is the preoperative CT scan as it allows for proper mapping and surgical planning. Magnetic resonance imaging (MRI) can also be helpful in distinguishing between inflammation and malignancy as well as allowing better visualization of soft tissue structures (thyrohyoid membrane, paralaryngeal space, true and false vocal cords). The information gathered provides guidance with regards to the extra care needed to avoid damage to neighboring structures [1].

After successful excision, anesthetic problems are extremely rare. The remainder of the management involves recovery and ruling out neoplastic lesions. The association between laryngeal saccular cyst and carcinoma means that histopathology studies are vital in excluding such tumors [2].

## Conclusion

Huge laryngeal saccular cysts demand proper anesthetic and surgical management planned in advance. Above all, the anesthetic part includes preparation for difficult airway and skills to deal with what otherwise would be unexpected. For the surgical part, the approach must be considered carefully as it is directly related to the optimal visualization and thorough mobilization of the cyst. Once these two areas are secured, post-operative airway problems are negligible.

## Consent

Written informed consent was obtained from the patient for publication of this case report and any accompanying images. A copy of the written consent is available for review by the Editor-in-Chief of this journal.

## Competing interests

The authors declare that they have no competing interests.

## Authors' contributions

IA and AS faced the clinical situation, made notes of it and gathered the data presented. All the authors contributed to the literature search and writing and reading of the manuscript. All authors read and approved the final manuscript.
